# P-995. Trends in antibiotic prescription rates among the Utah Medicaid population: a 4-year analysis

**DOI:** 10.1093/ofid/ofaf695.1194

**Published:** 2026-01-11

**Authors:** Tariq Mosleh, Devin beard, Angela Weil, Christina Radloff, April Clements, Sarah Rigby, Susan cheever, Karim Khader, Isabelle Stevenson

**Affiliations:** Utah Department of Health and Human Services, SLC, UT; Utah DHHS, SLC, Utah; Utah DHHS, SLC, Utah; Utah DHHS, SLC, Utah; Utah DHHS, SLC, Utah; Utah DHHS, SLC, Utah; Utah DHHS, SLC, Utah; University of Utah, Salt Lake City, Utah; University of Utah, Salt Lake City, Utah

## Abstract

**Background:**

Antibiotic overuse contributes to antimicrobial resistance and negative patient outcomes, including *Clostridioides difficile* infections and adverse drug events. Overall antibiotic prescribing in Utah is lower than the national rate, but it is unknown whether the Utah Medicaid population follows similar trends. The purpose of this study is to characterize patient demographics among the Utah Medicaid population and compare prescribing trends among this group against the general population in Utah and the US.

**Figure:**
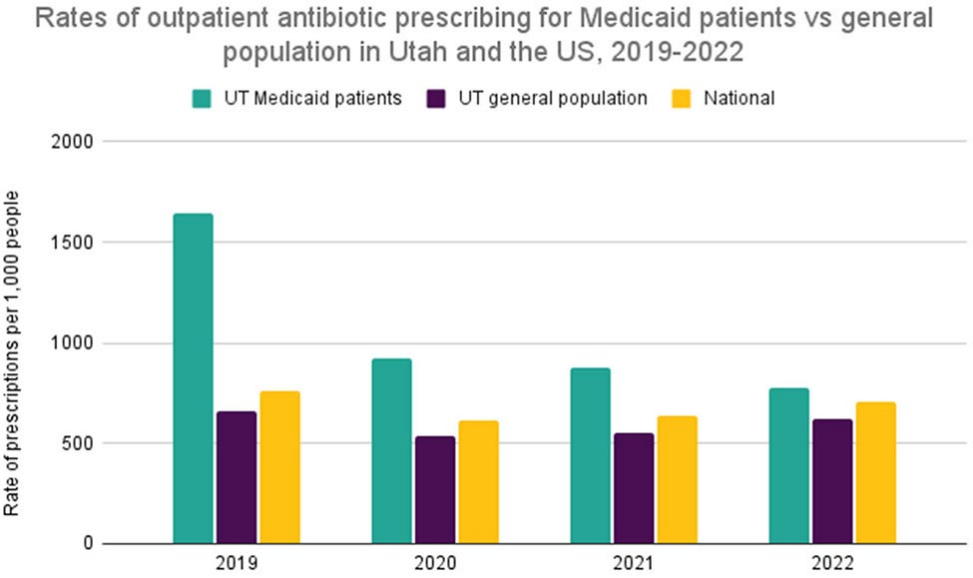
Rates of outpatient antibiotic prescribing for Medicaid patients vs general population in Utah and the US, 2019-2022

**Methods:**

This descriptive study analyzed Utah Medicaid outpatient antibiotic prescription claims from January 2019 to July 2023. Descriptive statistics were assessed for patient demographic data. Analysis was conducted using R.

**Results:**

We analyzed a total of 1,812,796 antibiotic pharmacy claims. Oral antibiotics constituted 93% of all prescriptions. The average patient age at prescription fill was 29.6 years, and the male-to-female ratio was 57:43. Overall, antibiotic prescription rates decreased 55% across the 4.5 year period. The highest monthly rates of antibiotic prescription fills occurred in 2019 (137 per 1,000) and decreased by 43% in 2020 (78 per 1,000). The Utah Medicaid population also had more antibiotics prescribed than the general Utah population and the US across all years with available data. Medicaid recipients in 2019 received 2 times more antibiotics than Utah's general population and the national level (2.48 more and 2.15 more, respectively). For the following years, rates remained higher for the Medicaid population ranging from 1.10-1.7 times more prescriptions.

**Conclusion:**

Overall, antibiotic prescription rates decreased across the study period; however, the Utah Medicaid population had more antibiotics prescribed than the general Utah population and the US across all years with available data. Conclusions regarding antibiotic prescription rates may be confounded by the COVID-19 pandemic, increased stewardship efforts, and the Medicaid expansion in January 2020, which may have led to healthier enrollees and artificially lowered prescription rates. Future directions include understanding and addressing factors driving antibiotic prescribing disparities among vulnerable populations.

**Disclosures:**

Karim Khader, PhD, bioMerieux: Grant/Research Support|Merck: Grant/Research Support

